# Spatial and Temporal Single-Cell Profiling of RNA Compartmentalization in Neurons with Nanotweezers

**DOI:** 10.1021/acsnano.5c02056

**Published:** 2025-05-06

**Authors:** Annie Sahota, Binoy Paulose Nadappuram, Zoe Kwan, Flavie Lesept, Jack H. Howden, Suzanne Claxton, Josef T. Kittler, Michael J. Devine, Joshua B. Edel, Aleksandar P. Ivanov

**Affiliations:** † Department of Chemistry, 4615 Imperial College London, Molecular Science Research Hub, London W12 0BZ, United Kingdom; ‡ Department of Pure and Applied Chemistry, 3527 University of Strathclyde, Glasgow G1 1BX, United Kingdom; § Department of Neuroscience, Physiology and Pharmacology, 4919 University College London, Gower Street, London WC1E 6BT, United Kingdom.; ∥ Kinases and Brain Development Lab, 376570 The Francis Crick Institute, 1 Midland Road, London NW1 1AT, United Kingdom; ⊥ Mitochondrial Neurobiology Lab, The Francis Crick Institute, 1 Midland Road, London NW1 1AT, United Kingdom; # Department of Clinical and Movement Neurosciences, UCL Queen Square Institute of Neurology, University College London, London WC1N 3BG, United Kingdom

**Keywords:** single-cell, nanotweezer, nanobiopsy, neuron, RNA, synaptic plasticity

## Abstract

Emerging techniques for mapping mRNAs within the subcellular compartments of live cells hold great promise for advancing our understanding of the spatial distribution of transcripts and enabling the study of single-cell dynamics in health and disease. This is particularly critical for polarized cells, such as neurons, where mRNA compartmentalization is essential for regulating gene expression, and defects in these localization mechanisms are linked to numerous neurological disorders. However, many subcellular analysis techniques require a compromise between subcellular precision, live-cell measurements, and nondestructive access to single cells in their native microenvironment. To overcome these challenges, we employ a single-cell technology that we have recently developed, the nanotweezer, which features a nanoscale footprint (∼100 nm), avoids cytoplasmic fluid aspiration, and enables rapid RNA isolation from living cells with minimal invasiveness. Using this tool, we investigate single-cell mRNA compartmentalization in the soma and dendrites of hippocampal neurons at different stages of neuronal development. By combining precise targeting with sequential sampling, we track changes in mRNA abundance at dendritic spine regions of the same neuron, both before and after stimulation. This minimally invasive approach enables time-resolved, subcellular gene expression profiling of the same single cell. This could provide critical insights into polarized cells and advance our understanding of biological processes and complex diseases.

## Graphical abstract

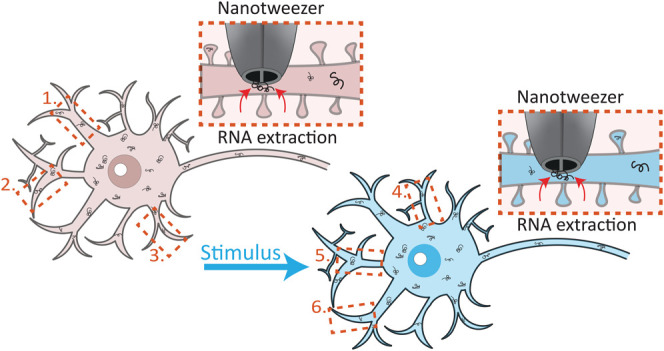

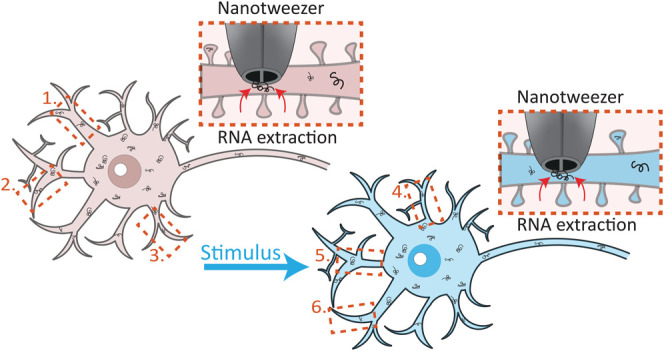

## Introduction

Single-cell transcriptomics has transformed our understanding of the complexity and heterogeneity of biological systems. However, accessing the complete transcriptome of polarized cells such as neurons is challenging due to difficulties in isolating the neurites and intricate structures of the cell.^1^ This means we often lack detailed transcriptomic information on neuronal processes despite their critical roles in brain function. Messenger RNA (mRNA) compartmentalization plays a crucial role in regulating gene expression in neurons, which is essential for neuronal homeostasis and represents the primary mechanism of protein localization.^2–4^ Defects in RNA regulation have been implicated in many neurological disorders, including Alzheimer’s disease,^5,6^ amyotrophic lateral sclerosis,^7–9^ and Fragile X syndrome.^10,11^ Thus, deciphering the dynamics of mRNA and its compartmentalization is critical to understanding neuronal development, function, and disease. There is, therefore, a growing need to complement single-cell transcriptomics with techniques that reveal the subcellular distribution of transcripts to fully understand the functional implications of specific mRNAs in single cells. However, many techniques that analyze the subcellular contents of neurons are limited in achieving the necessary spatial and temporal resolution for deciphering compartmentalized processes in living cells.

Although imaging techniques, such as those based on fluorescent in situ hybridization (FISH)^12–16^ and live-cell imaging,^17–19^ have opened up possibilities to access the spatial distribution of transcripts in neurons, isolating biomolecules from specific cellular locations would enable the molecular basis of subcellular compartments to be defined while minimizing the delivery of sequence-specific exogenous probes into cells and avoiding cell fixation. The physical isolation of axons and dendrites for downstream transcriptomic analysis has been one way of achieving this. Neuronal compartments have been isolated using various techniques, including microdissection,^14,20–22^ microporous membranes,^15,23^ or microfluidic chambers.^24^ However, these methods are destructive, leading to a loss of interconnection and only allowing for static transcriptomic analyses. Compartments may also be pooled for bulk downstream analysis, losing further contextual and spatial information about the cells.

The advent of various single-cell sampling and nanobiopsy tools, which involve the nondestructive insertion of sampling probes into live single cells to analyze their intracellular makeup, offer potential solutions to these problems.^25^ These techniques include nanopipettes,^26–31^ FluidFM,^32–36^ nanostraws,^37^ nanoneedles,^38^ and nanotweezers,^39,40^ which have the potential to study the subcellular compartmentalization of single cells without destroying the cell or losing important contextual information. Until recently, targeted single-cell sampling has been limited to single time point analyses. For example, nanobiopsy has been used to study the compartmentalization of mRNAs in neuronal cells, but only static analyses from pooled samples were performed.^28^ This method involved the aspiration of cytoplasmic fluid to access mRNA in the neurites and soma of neuronal cells. Time-dependent measurements on the same live cells using FluidFM-based Live-seq have undoubtedly extended the capabilities of these techniques, where dynamic sampling was performed to track the transcriptome of living cells.^36^ This was achieved by removing between 0.2–3.5 pL of the cellular volume at each time point. Similarly, nanobiopsy-based SICM has enabled longitudinal profiling of cancer cells to measure radiotherapy and chemotherapy treatment responses, where up to ∼200 fL was removed from cells at each time point.^27^

The nanotweezer, a technique that we previously reported on, offers a unique way to sample from living cells by directly trapping biomolecules via dielectrophoresis (DEP), negating the need to remove large volumes from cells.^39^ High electric field strengths (≤10^28^ V^2^ m^–3^) can be generated to trap a range of biomolecules, including DNA, RNA, and organelles, in their native cellular environments. Compared to other techniques that involve the aspiration of cytoplasmic fluid and alterations in cellular volume, the nanotweezer can concentrate nucleic acids and organelles within 300 nm of the nanotweezer tip, enabling direct isolation of biomolecules with high spatial resolution. This makes it a highly promising tool for minimally invasive subcellular sampling from precise regions of living neurons. In recent work, the sampling ability of the nanotweezer was exploited to detect the presence of differentially localized mRNAs in both healthy and failing cardiomyocytes, demonstrating that gene expression could be detected in different locations of these cells.^40^

Here, we present dynamic subcellular mRNA measurements in living single neurons using nanotweezers, enabling localized single-cell gene expression tracking over time. Gaining access to subcellular neuronal compartments and performing live-cell measurements are both essential for capturing single-cell responses and elucidating mRNA dynamics and plasticity. However, preserving the integrity and viability of sensitive cell types, such as primary neurons, is critical for studying mRNA localization in real-time. We demonstrate the extraction of RNA from multiple precise compartments of primary neurons, including the soma, axons, dendrites, and synaptic regions. Multiple extractions from the same live cell were possible due to the minimally invasive nature of the technology, allowing for mRNA compartmentalization to be studied without impacting cell viability. Furthermore, sequential sampling of the same cell following synaptic stimulation allowed for dynamic changes in mRNA localization to be detected with high spatial resolution, thus enabling a time-dependent exploration of single-cell and intracellular responses.

This minimally invasive method to study local mRNA dynamics in living cells indicates a great potential for uncovering the inherent variability of single cells and providing true single-cell gene expression responses.

## Results and Discussion

### RNA Profiling of Live Neurons Using the Nanotweezer

The nanotweezer was used to perform single-cell RNA sampling from different compartments of live hippocampal neurons (Figure 1a). The nanotweezer comprised a tunable double barrel nanopipette with a total tip size of ∼100 nm (Figure 1b­(i)) to access the compartments of living cells. Pyrolytic carbon deposition in the nanopipette formed two carbon nanoelectrodes separated by a nanometric gap (Figure 1b­(i)). The nanotweezer was used to trap and isolate RNA from neuronal subcompartments by dielectrophoretic trapping. This was initially validated using SYTO RNASelect to label intracellular RNA (Figure 1d­(ii),(iii)). Briefly, a micromanipulator, monitored by bright-field imaging, was used to maneuver the nanotweezer to the region of interest within a single neuron, followed by gentle insertion of the nanotweezer tip into the cell (Figure 1c­(i),d­(i)). The application of an alternating (AC) voltage enabled RNA to be trapped and concentrated at the nanotweezer tip (Figure 1c­(ii),d­(ii)). The nanotweezer was then removed from the cell to extract the trapped RNA, confirmed by the presence of fluorescence on the tip after removing the nanotweezer from the cell (Figure 1d­(iii)), and the isolated RNA was taken forward for downstream RT-qPCR analysis (Figure 1c­(iii)). Consistent extraction of RNA from the cytoplasm highlighted the potential to analyze gene expression from subcellular compartments of living neurons using nanotweezer sampling.

**Figure 1:**
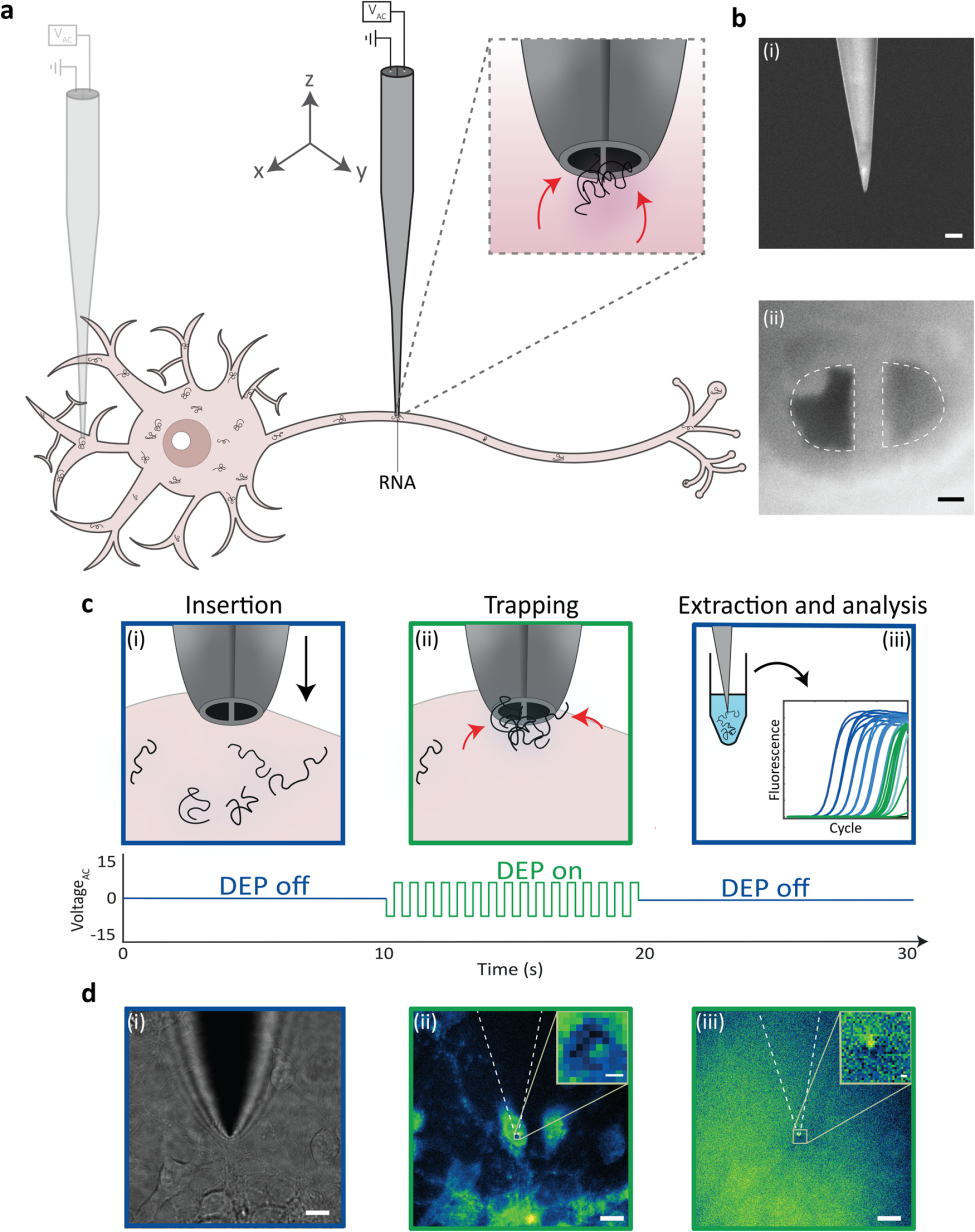
Trapping and extraction of RNA from live neurons. (a) Schematic of dielectrophoretic trapping of RNA from neurons using the nanotweezer. (b) Scanning electron microscopy images of the nanotweezer tip. Side view (i) scale bar = 500 nm. Tip view (ii) scale bar = 20 nm. (c) Schematic of the RNA extraction process. The nanotweezer is inserted into a subcellular compartment of a live neuron (i), application of an AC voltage causes RNA molecules to become dielectrophoretically trapped at the nanotweezer tip (ii), the nanotweezer is removed from the cell to extract the trapped RNA for retrieval and downstream analysis (iii). (d) RNA trapping procedure with cells labeled with SYTO RNASelect. The nanotweezer is inserted into a neuron using bright field imaging to guide the positioning (i). Applying an AC field causes dielectrophoretic trapping of RNA at the nanotweezer tip (ii). The nanotweezer is removed from the cell, and the presence of isolated RNA at the nanotweezer tip is confirmed by fluorescence (iii). Inset images show the position of the nanotweezer tip. Scale bars = 10 μm, inset scale bars = 500 nm.

Neurons are highly polarized cells made up of distinct compartments that are essential for neuronal function (Figure S1). To gain a comprehensive understanding of the processes in neurons via single-cell sampling, multiple precise RNA extractions need to be taken from cells to obtain a representative view of their compartmentalized expression profiles. However, primary neurons are particularly sensitive cells, made up of intricate neurites and substructures that are highly vulnerable to damage. Ensuring these intricate structures can be accessed without cell destruction is paramount for accurate gene expression mapping in live cells. To explore this possibility, we extended our nanobiopsies to access multiple compartments of hippocampal neurons to measure the RNA content of defined subcellular regions. Specific targeting was achieved by adjusting the *z*-position of the objective for each cellular region before nanotweezer sampling. This ensured that the region of interest was in the plane of focus and accounted for the differences in thickness throughout the cell. Nanobiopsies were performed on label-free (Figure S2a) and EGFP-expressing neurons (Figure S2b). The nanotweezer could repeatedly sample from multiple locations of the same living neuron, including the soma, axon, and dendrites (Figure 2a). The integrity of the neurons was also maintained following multiple biopsies, confirmed by the continued observation of intact neuronal structures and the retention of EGFP (Figure S2b). Up to eight biopsies in total from the same cells were performed on two consecutive days without affecting cell viability (Figure S3), highlighting the potential for the tool to be used to study compartmentalized and time-resolved gene expression in living cells via minimally invasive sequential extractions from the same cell.

**Figure 2:**
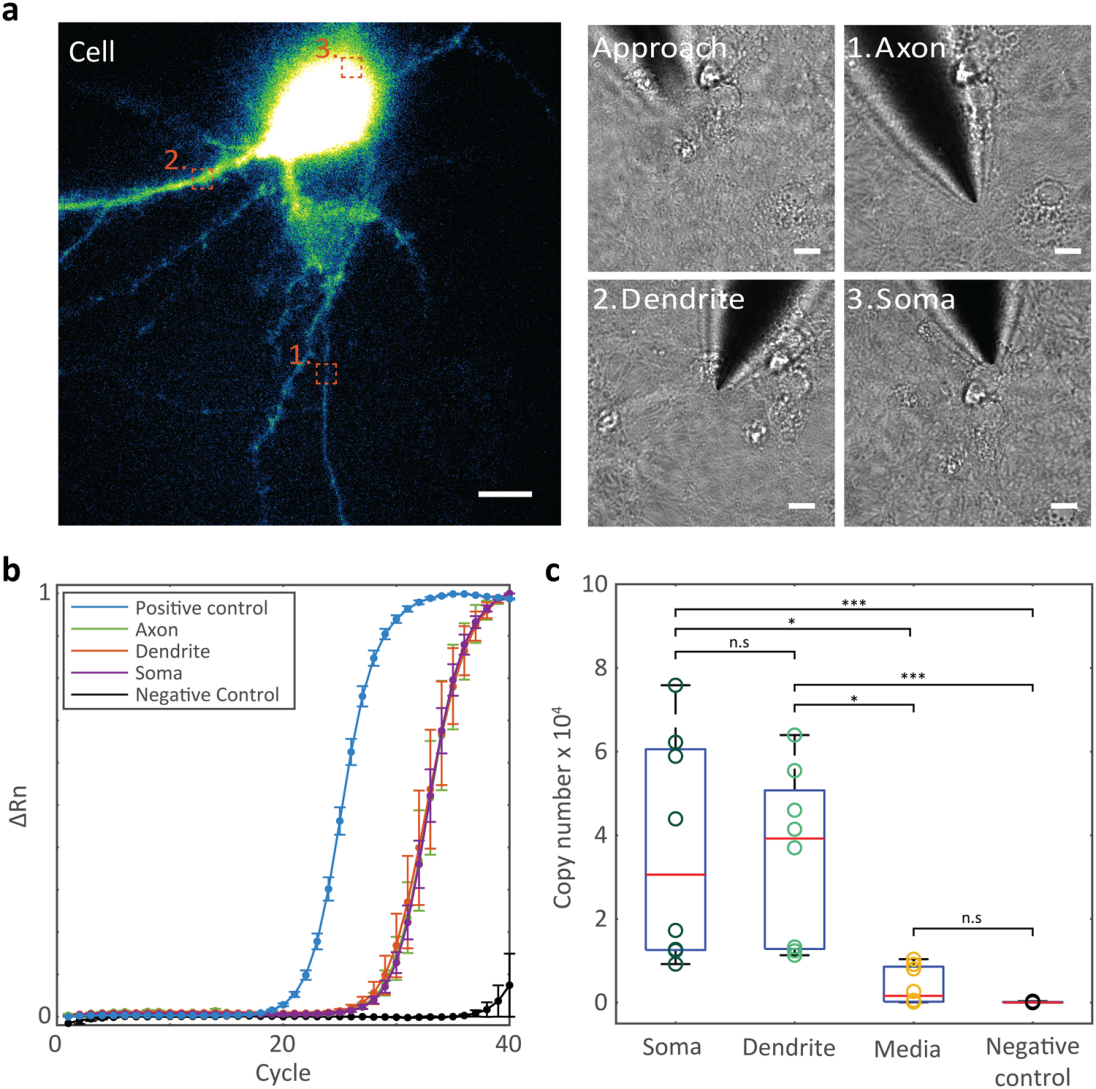
Detection of RNA from subcellular locations of living neurons. (a) Fluorescent image (i) of an EGFP-filled hippocampal neuron targeted for multiple DEP extractions from the axon (1), dendrite (2), and soma (3) of the same cell. Orange squares illustrate the nanobiopsy locations. Corresponding bright field images (ii) of the nanotweezer during approach to the cell, axonal nanobiopsy, dendritic nanobiopsy, and somata nanobiopsy. Scale bars = 10 μm. (b) RT-qPCR amplification of 18S rRNA from axonal, dendritic, and somata DEP nanobiopsies from hippocampal neurons. Data presented as mean ± s.e.m (*n* = 3). (c) Boxplot of 18S rRNA copy numbers extracted from individual DEP nanobiopsies from the soma, dendrites, and extracellular media, compared to RT-qPCR (no template) negative controls (*n* = 8). Statistical significance was determined by the Kruskal–Wallis test with Dunn’s multiple comparison test (*P < 0.05, ***P < 0.001, n.s = not significant). Summary statistics for boxplot: center = median; bounds of box = IQR 25th and 75th percentile; whiskers = minimum and maximum within 1.5 IQR.

RT-qPCR was performed directly on the samples to investigate whether sufficient RNA was isolated from individual DEP nanobiopsy samples for gene expression analyses. 18S rRNA was successfully detected in extractions from all locations using RT-qPCR (Figures 2b and S4 and Table S1), exhibiting the detection of gene expression in neurons with subcellular resolution. The RNA molecules detected were shown to derive from the cell itself, demonstrated by significantly larger copy numbers of 18S rRNA detected in DEP nanobiopsies from neurons compared to the application of DEP in the cells’ culture medium and RT-qPCR negative controls. This confirmed that the detected RNA was extracted from the targeted neuronal compartments. The detection of some RNA from applying DEP in the cell culture medium may be attributable to extracellular RNA from exocytosis. Due to the nanotweezer requiring contact with the cells’ medium during the biopsy procedure, the isolation of RNA from the cell culture medium cannot be fully avoided. However, it was shown to be minimal overall compared to RNA isolated from the cells.

### Spatially Resolved Single-Cell Nanobiopsy for mRNA Compartmentalization

To study mRNA compartmentalization in the same single cell, the distribution of microtubule-associated protein 1A (MAP1A) mRNA, an mRNA that has been shown to localize to the dendrites of neurons,^13,20,21,41,42^ was measured as a proof-of-concept study to detect differential gene expression using the nanotweezer platform. MAP1A is a brain-specific microtubule-associated protein important for microtubule stability and assembly.^43^ As with other microtubule-associated proteins, it plays an important role in neurogenesis.^44^ As neurons mature, their processes extend out of the soma as axons or dendrites and synapses are established. Significant gene expression and protein production changes occur in the cells to account for this development, including the upregulation of MAP1A in the dendrites.^45,46^ To explore the dendritic localization of MAP1A in single neurons, multiple biopsies were performed in the soma and proximal dendrites of hippocampal neurons from days-in vitro (DIV) 3 to DIV 11 (Figure 3a). The proximal dendrites were targeted to allow for simple targeting of the dendrites and consistency between cells, particularly as the abundance of transcripts may change with increasing distance from the soma. The MAP1A copy numbers of the soma and dendrites of the same cell were compared to enable accurate single-cell expression analyses (Figure 3b and Table S2). Variation between different nanobiopsies from the same compartment of the same cell was observed. For example, for a typical cell (Cell 1 in Table S2), the normalized MAP1A copies from the soma and dendrites were 7.1 × 10^–4^ ± 1.1 × 10^–4^ and 4.1 × 10^–4^ ± 1.8 × 10^–4^, respectively (mean ± s.d). The variation between samples was expected, given that each extraction was performed in a different region of the compartment using a highly localized trapping volume of ∼300 nm from the nanotweezer tip. Intracellularly, this variation may be due to local, native fluctuations in mRNA abundance and dynamics within a specific compartment, where mRNA abundance is unlikely to be fully uniform throughout a compartment at the high spatial resolution that the nanotweezer achieves. The time between nanobiopsies taken from the same cell (8 ± 2 min) and the number of RNA extractions per cell (2 to 8) may also lead to some short-term gene expression fluctuations.

**Figure 3:**
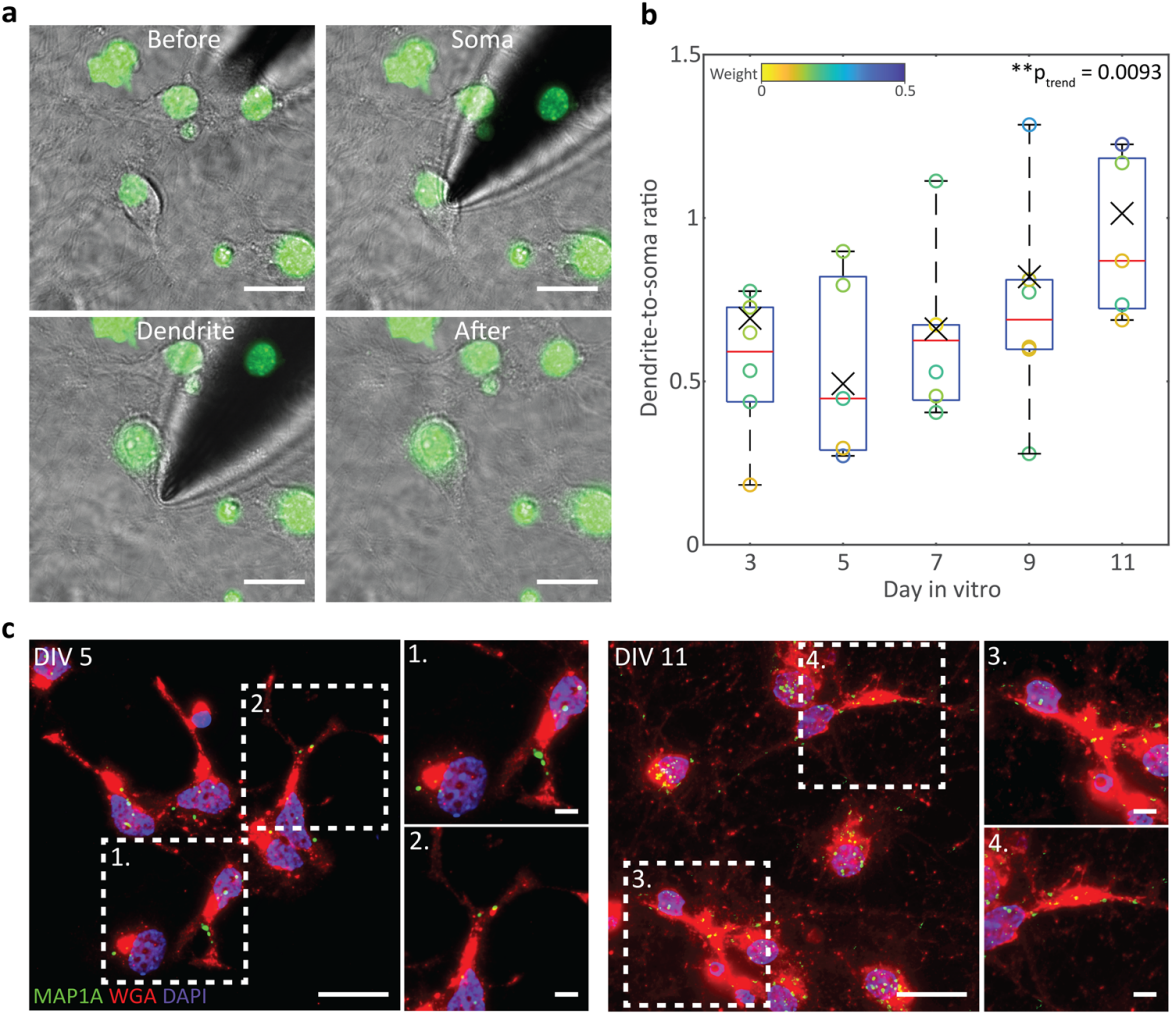
Detection of mRNA localization. (a) Hippocampal neurons before, during, and after DEP nanobiopsies from the soma and dendrite of the same cell. Nuclei were stained with NucSpot Live 488 (green). Scale bars = 20 μm. (b) Boxplots of the dendrite-to-soma ratios of MAP1A expression per cell at different days in vitro (*n* = 6 cells). Cells were weighted based on the number of nanobiopsies conducted per cell (2 to 8). The color bar represents the weights applied to individual cells. Statistical significance was determined by a one-way ANOVA using the weighted means followed by a linear trend multiple comparison test (**p < 0.01). Summary statistics for boxplot: center = median; bounds of box = IQR 25th and 75th percentile; whiskers = minimum and maximum within 1.5 IQR; *X* = weighted mean. (c) Maximum *z*-projection images of smFISH of DIV 5 and DIV 11 hippocampal neurons. A probe for MAP1A mRNA (green) was incorporated, and cells were counterstained with WGA (red) to label the plasma membrane and DAPI (blue) to stain the nuclei. Dashed boxes illustrate dendritic regions of interest. Scale bars = 20 μm, zoomed region scale bars = 5 μm.

A linear trend analysis was performed following a one-way ANOVA to determine whether there was a significant directional trend in the dendrite-to-soma ratios of cells between DIV 3 to DIV 11. A significant increasing trend in the dendrite-to-soma ratios was observed overall between these time points (*P* = 0.0093). The results indicate a progressive shift, where MAP1A mRNA localizes more in the dendrites during development. Although not impacting the overall trend, DIV 3 did not show the same pattern as the other DIV. DIV 3 represents a very early stage of neuronal development, where projections from the soma have begun, but the identity of the dendritic processes may be unclear until ∼DIV 5, when their growth rates increase significantly.^47^ This was demonstrated by immunocytochemistry (Figure S5), where very short processes were present at DIV 3 with no, or very few, higher-order dendrites. A significant (*P* < 0.0001) increase in dendritic growth was observed between DIV 3 and 5, and the visual identity of the dendritic processes becomes more apparent from MAP2 labeling. The deviation from the observed trend in MAP1A localization at DIV 3 may be due to the lack of fully formed dendrites and unestablished RNA regulation. The overall detection of MAP1A in the dendrites and increasing trend in MAP1A dendrite-to-soma ratios with neuronal development is consistent with other studies based on in situ hybridization,^48^ compartment isolation followed by RNA sequencing (RNA-Seq),^14,42^ and multiplexed error-robust FISH (MERFISH) that compared two DIV time points.^13^ To validate this localization behavior further, single-molecule FISH (smFISH) was performed on DIV 5 and DIV 11 hippocampal neurons (Figures 3c and S6). MAP1A mRNA was observed in the dendrites of both DIV 5 and DIV 11 hippocampal neurons, supporting the detection of MAP1A in dendritic nanobiopsy samples. The fluorescent spots corresponding to MAP1A mRNA were observed more significantly in the dendrites at DIV 11. Thus, the results from smFISH confirmed the sampling capabilities of the nanotweezer for analyzing localized transcripts in single neurons.

### Dynamic RNA Profiling from Precise Locations of Live Single Neurons

The nanotweezer offers a simple alternative to smFISH for detecting RNA localization at single time points, providing information from live cells with minimal cellular damage instead of cell fixation in FISH. However, the true benefit of the technology lies in its potential to make sequential measurements in the same live cell to monitor true single-cell responses. Given the inherent heterogeneity of single cells, tracking a cell’s gene expression from its ground state is essential to fully understanding cellular dynamics, disease progression, or treatment responses. The nanotweezer’s ability to sample from highly localized regions of single cells without affecting cell viability showed promise for single-cell tracking with high intracellular spatial resolution.

To explore the possibility of profiling transcripts in precise subcellular regions of the same living cell over time, we chose a model of *N*-methyl-d-aspartate receptor (NMDAR) activation to measure short-term changes in transcript abundance in the synaptic regions of the same neurons before and after stimulation (Figure 4a). Hippocampal neurons were plated on gridded dishes to enable the same neuron to be located after stimulation and biopsied at DIV 14–15–a time point at which mature dendritic spines would be present (Figure 4b­(i),(ii)) for effective synaptic plasticity. The nanotweezer was then precisely targeted to the base of the dendritic spines (Figure 4c­(i)), defined as an area within a dendrite where the projection of a spine begins, and biopsied to extract the RNA from this precise location (Figure 4c­(ii)). While other sampling techniques may face challenges with minimally invasive access to highly precise cellular subcompartments such as the dendritic spines due to their larger probe size or requirement to aspirate cytoplasmic volumes containing the molecules of interest, the nanotweezer traps molecules directly from the cytoplasm which, when combined with careful manipulation, enables tight control over targeted regions. This was indicated by successful sequential biopsies from the base of the dendritic spines from the same cells without a loss of cell or spine integrity during the procedure (Figures 4c­(iii) and S7).

**Figure 4:**
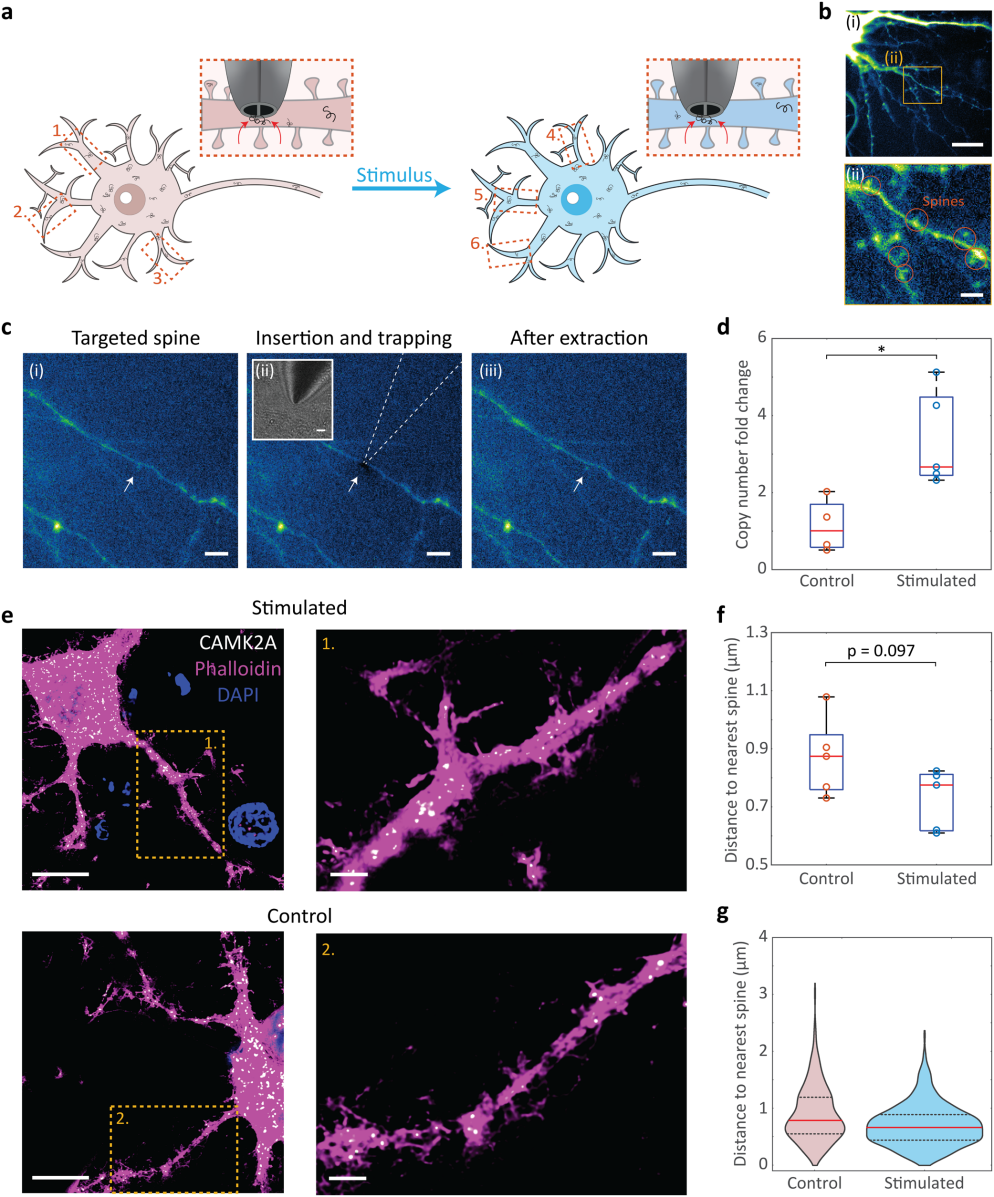
Spatially and temporally resolved RNA measurements from the same cell during stimulation. (a) Schematic of the time-resolved single-cell mRNA response study. The base of dendritic spines was targeted by the nanotweezer for local RNA extraction. In its original cultured state, 2–3 nanobiopsies were performed at different dendritic spines of the same cell. Chemical stimulation was then applied to the cell using NMDA/glycine, and 2–3 further nanobiopsies were performed at the spines of the same live cell after stimulation. (b) Representative fluorescent images of EGFP-filled DIV 14 hippocampal neurons (i), highlighting one representative dendritic region (square) zoomed (ii) to show the dendritic spines (circles). Scale bars = 10 μm (i) and 5 μm (ii). (c) Fluorescent images of the dendritic spine nanobiopsy procedure. A dendritic spine is targeted in the cell (i), the nanotweezer is inserted into the cell at the base of the targeted dendritic spine, and an AC voltage is applied to extract RNA from this precise location (ii), the nanotweezer is removed from the cell to extract the RNA, and the cell and spine remain intact (iii). Inset: Corresponding bright field image of nanotweezer position in the cell. White arrows represent the location of the targeted spine. Scale bars = 5 μm. (d) Change in CAMK2A expression at the dendritic spines of the same neurons in stimulated vs control cells following DEP nanobiopsies (*n* = 5 cells). Statistical significance was determined by an unpaired *t* test (*p < 0.05). Summary statistics for boxplot: center = median; bounds of box = IQR 25th and 75th percentile; whiskers = minimum and maximum within 1.5 IQR. (e) Maximum *z*-projection smFISH images of neurons with example dendritic regions highlighted (orange box) in stimulated (top) and control (bottom) cells. A probe for CAMK2A (green) was incorporated to visualize CAMK2A mRNA. Cells were counterstained with phalloidin (magenta) as a neuronal and dendritic spine marker and with DAPI (blue) to stain the nuclei. Scale bars = 20 μm, zoomed scale bars = 2 μm. (f) Quantification of CAMK2A mRNA distances to the nearest spine from smFISH (*n* = 5 cells). Statistical significance was determined by an unpaired *t* test. (g) Violin plot of total CAMK2A mRNA distances to the nearest spine measured over 5 cells (*n*_stimulated_ = 372, *n*_control_ = 197). The red line on the violin plot represents the median, and the dashed lines represent the 25th and 75th percentile IQR.

Activation of NMDARs can induce long-term potentiation (LTP), a form of synaptic plasticity that involves the continued strengthening of synapses for enhanced neuronal transmission, which is suggested to be involved in learning and memory formation.^49,50^ To assess if we could detect changes in mRNA dynamics in live cells using our technology and this model of NMDAR activation, two to three biopsies were taken from the base of the dendritic spines from the same cell before and after chemical stimulation with *N*-methyl-d-aspartic acid (NMDA) and glycine, agonists for NMDARs. The same procedure was applied to control cells in the absence of an agonist. The time between each dendritic spine nanobiopsy under the same condition (unstimulated, stimulated, or control) was 10 ± 2 min, which, in addition to minor natural fluctuations in RNA abundance, may lead to some variation between sequential nanobiopsy events in the same cell. The expression of Calcium/Calmodulin Dependent Protein Kinase II Alpha (CAMK2A) mRNA, a dendritically localized mRNA that encodes for a subunit of the CAMKII protein involved in regulating excitatory transmission and LTP,^51,52^ was analyzed using RT-qPCR. To derive true single-cell transcriptional responses to NMDAR activation, overall CAMK2A mRNA abundance from the same cell before and after treatment was compared (Figure 4d and Table S3). An increase in CAMK2A mRNA abundance at dendritic spine regions was shown with stimulation compared to control cells. This is consistent with previous work showing an accumulation of CAMK2A mRNA near the dendritic spines following chemical LTP,^18,53^ validating the nanotweezer to measure single-cell responses.

A comparison of nanotweezer sampling was then made to smFISH (Figure 4e). As smFISH cannot measure dynamic single-cell responses, a comparison of CAMK2A mRNA in fixed stimulated cells vs fixed control cells was made. To determine the localization of CAMK2A mRNAs to dendritic spine regions, the distance of individual CAMK2A molecules to the base of the nearest spine was measured in the dendritic regions of cells (Figure 4f). No significant difference between the association of CAMK2A mRNAs to dendritic spine regions in stimulated and control cells was observed from smFISH results. Nevertheless, an overall tendency of CAMK2A toward the spines (Figure 4g) and lower median distances in stimulated cells was shown, which may suggest similar trends of increased spine localization.

Dielectrophoretic intracellular extractions from the same live cells provide an advantage over smFISH measurements because they provide live-cell responses. In contrast, cells are fixed at a singular time point in smFISH. This could explain the difference in significance levels between the two methods despite the same number of cells measured. The nanotweezer allowed for the stimulated state of a cell to be compared to its ground state and, therefore, accounts for the variation between single cells. FISH, however, can only obtain an overall effect from cell populations, where cells will have varying gene expression at specific time points, meaning that the transcriptomic change of a cell from a particular stimulus may not be captured. The nanotweezer sampling results described here may provide a more accurate depiction of gene expression changes in single cells, unmasking the heterogeneity between ostensibly similar cells. This highlights the importance of studying single-cell responses; the advancement in single-cell sampling capabilities described herein could be used to further our understanding of RNA regulation in single cells and uncover rare subcellular mechanisms in cell populations.

## Conclusions

Accessing the precise subcellular compartments of polarized cells is essential to understanding RNA regulation in health and disease. Given the heterogeneity of single cells, there is a vital need to track gene expression from live cells to measure the gene expression trajectory of single cells in their spatial context. In this work, we demonstrate precise gene expression tracking of the subcellular compartments of live cells by combining the high intracellular spatial resolution of the nanotweezer with sequential sampling.

Through precise sampling of neurons, different subcellular compartments of the same cell were accessed for local mRNA isolation and analysis without affecting the viability of this sensitive cell type. This subcellular targeting is particularly critical for neurons, where much of their protein production machinery is decentralized to distal locations in the cell. We, therefore, provide a simple method to measure differential mRNA regulation in single cells with high subcellular resolution. To further exploit the technology’s precise targeting and minimally invasive nature, the base of the dendritic spines was targeted to extract RNA from postsynaptic regions of the same neurons before and after activation of the synapses, a mechanism tightly regulated for neuronal homeostasis. This work has advanced the spatial resolution offered by subcellular sampling techniques, where precise subregions within the dendrites were targeted without destroying the intricate neuronal structures. We also demonstrated a time-dependent study, where changes in mRNA abundance at these subcompartments in a neuron’s unstimulated ground state could be compared to the same cell’s stimulated state. This opens up novel opportunities for measuring RNA dynamics in the substructures of living cells to allow for true single-cell responses to treatments or stimuli to be derived.

The nanotweezer’s highly localized trapping region from the tip (300 nm) is extremely desirable for detecting RNA fluctuations in intricate regions and subregions of single cells and sets it apart from other subcellular sampling techniques that require the aspiration of large cellular volumes from whole cells. However, the amount of RNA extracted from these localized regions using the nanotweezer is currently insufficient for reliable transcriptomic analyses. A balance between improving the nanotweezer’s DEP trapping ability while still maintaining its high subcellular precision and minimal impact on the cell is required. This, combined with the continual development of methods for low-input RNA-Seq, could enable transcriptomic analyses of individual nanotweezer extractions and allow for highly compartmentalized transcriptome tracking of living cells. Additionally, the manual operation of the nanotweezer limits the spatial and temporal resolution between multiple extractions of the same cell. Integrating the nanotweezer with SICM would greatly enhance this,^54^ enabling more control over the extraction position and allowing for the same extraction location to be returned to and tracked.

Emerging tools with high spatial and temporal resolution may lead to a more detailed understanding of the molecular mechanisms involved at the neuronal level and help elucidate the key RNA regulatory drivers of neurodegeneration, providing opportunities for possible therapeutic interventions. In this context, the nanotweezer provides a promising tool to probe the localized RNA dynamics of neurons in their native microenvironment with minimal alterations. Further developments to automate the procedure and improve the DEP trapping ability would enhance the throughput of the technique, allowing for a comprehensive panel of transcripts and their dynamics in precise subcompartments to be studied. With these developments, we anticipate the discovery of rare transcripts in defined single-cell compartments and under different cellular states, contributing to temporal subcellular transcriptomics, local treatment monitoring, and longitudinal disease profiling.

## Materials and Methods

### Primary Neuron Culture

Hippocampal primary neuronal cultures were isolated from E18 rats and plated at a density of 25,000 cells/cm^2^ on 35 mm glass bottom dishes (Greiner Bio-One) precoated with 0.25 mg/mL poly-l-lysine hydrobromide (Sigma-Aldrich) overnight. Cells were attached overnight in Minimum Essential Medium (Gibco) supplemented with 10% horse serum (Gibco), 1 mM pyruvic acid (Sigma-Aldrich), and 0.6% glucose solution (Sigma-Aldrich). Cells were then cultured in Neurobasal (Gibco) supplemented with 2% B27 (Gibco), 1% Glutamax (Gibco), 0.6% glucose (Sigma-Aldrich), and 1% Penicillin-Streptomycin (Gibco) at 37 °C with 5% CO_2_. Half media changes were performed every 3–4 days.

For NMDAR activation experiments, neurons were plated on 35 mm gridded dishes (Ibidi) and maintained in Neurobasal Plus (Gibco), 2% B27 Plus (Gibco), 1% Glutamax (Gibco), 0.6% glucose (Sigma-Aldrich) and 1% Penicillin-Streptomycin (Gibco). NMDARs were activated based on a previously reported protocol.^55^ DIV 14–15 neuronal cultures were incubated in warm Mg^2+^-free buffer (126 mM NaCl, 2 mM CaCl2, 2.5 mM KCl, 5 mM HEPES, 11.1 mM glucose, 1 μM strychnine (Sigma-Aldrich), 20 μM bicuculline (Sigma-Aldrich), 0.5 μM tetrodotoxin (Abcam), pH 7.4) for 15 min, followed by application of 50 μM NMDA and 10 μM glycine in Mg^2+^-free buffer for 3 min and then replacement with conditioned medium for 5 min. Controls were treated with the same buffer in the absence of agonists.

### Labeling and Transfection

Fluorescent dyes were loaded onto cells immediately before imaging. The RNA of neurons was labeled by loading cells with 500 nM SYTO RNASelect (Invitrogen) for 20 min. Cells were then washed with warm PBS, and the labeling solution was replaced with maintenance media. The nuclei of cells were labeled by incubating cells with 1X NucSpot Live 488 (Biotium) for 10 min at 37 °C. For viability assessments, cells were incubated with 2 μM Calcein AM (Invitrogen) for 20 min at 37 °C. Cells were then washed with warm PBS, and the solution was replaced with maintenance media. For EGFP-filled neurons, neurons were transfected by lipofection 2 days before nanobiopsy experiments using Lipofectamine 2000 (Life Technologies) and mEGFP-C1 DNA (Addgene plasmid # 54759 from Michael Davidson; http://n2t.net/addgene:54759; RRID: Addgene_54759).

### DEP Nanobiopsy

Nanotweezers were fabricated, as reported previously.^39^ DEP nanobiopsy was performed on cultured neurons mounted on an inverted optical microscope (IX71, Olympus) with a 60× water-immersion objective (1.20 numerical aperture, UPLSAPO 60XW, UIS2, Olympus). Bright-field and fluorescent microscopy were used to guide the procedure. Cells were illuminated with a fiber-coupled 488 nm diode-pumped solid-state laser (Sapphire 488–50 CDRH, Coherent) coupled to the microscope via a TIRF module (cellTIRF, Olympus) with single-mode optical fiber. Images were acquired with a scientific complementary metal-oxide-semiconductor (sCMOS) camera (Marana 4.2B-11, Andor). The nanotweezer was mounted on a motorized stage using a micromanipulator (PatchStar, Scientifica) perpendicular to the cell culture dish on the imaging setup. Electrical contact with each electrode of the nanotweezer was achieved by inserting copper wires through each barrel of the pipet. The other ends of the copper wires were connected to a function generator (TG2000, TTi, U.K.), which was used to apply DEP to the nanotweezer. Using fluorescence or bright-field illumination, the subcellular region of interest was targeted. The nanotweezer was then positioned using *x*- and *y*-axis manipulators, followed by a *z*-approach and insertion into the cellular region of interest using the *z*-direction using bright-field imaging. DEP was applied for 10 s by applying an AC voltage between the electrodes (1 MHz, 15 *V*_peak‑to‑peak_) using the function generator to trap RNA at the nanotweezer tip. The nanotweezer was then retracted from the cell while maintaining the DEP force, and the trapped material was transferred to a PCR tube by pressing the tip into the tube containing 5 μL nuclease-free water. Trapped material was either used immediately for reverse transcription or stored at −80 °C for <1 month.

### qPCR Primers and Probes

All primers and probes used for qPCR are listed in Table S4. Primers and probes were designed using Primer-BLAST (https://www.ncbi.nlm.nih.gov/tools/primer-blast/), PrimerQuest (https://eu.idtdna.com/PrimerQuest/) or ordered commercially (Integrated DNA Technologies). For multiplexed qPCR, the compatibility of primer/probe combinations was validated using the OligoAnalyzer Tool (https://eu.idtdna.com/pages/tools/oligoanalyzer/) and Eurofins Genomics Oligo Analysis Tool (https://eurofinsgenomics.eu/en/ecom/tools/oligo-analysis/). For predesigned commercial primer/probe assays, a 2:1 primer:probe ratio was incorporated.

### RT-qPCR

Nanobiopsy samples were converted into cDNA using the iScript cDNA Synthesis Kit (Bio-Rad) using a thermal cycler (Techne TC- 3000). 1 μL reverse transcription enzyme was added alongside 4 μL iScript reaction buffer to individual samples for reverse transcription. All qPCR reactions were performed using a StepOnePlus Real-Time 96-well PCR system (Applied Biosystems) in MicroAmp optical strip 0.1 mL tubes (Applied Biosystems) or MicroAmp optical 0.1 mL 96-well plates (Applied Biosystems). For initial validation experiments (Figure 2), singleplex reactions were run on cDNA samples using SsoAdvanced Universal SYBR Green Supermix (Bio-Rad) over 40 cycles with the thermocycling and reaction conditions set according to the manufacturer’s protocol. A melt curve stage was run after the amplification protocol by increasing the temperature at a rate of 0.5 °C/s from 60 to 90 °C. All other experiments were performed as duplex reactions using PrimeTime Gene Expression Master Mix (Integrated DNA Technologies). A 2:1 primer: probe ratio (500:250 nM) in a final reaction volume of 20 μL was incorporated with thermocycling over 40–45 cycles according to the manufacturer’s protocol. All duplex reactions were run by multiplexing the target primers/probe with 18S primers/probe as the positive control. Copy numbers of all targets were acquired by the absolute quantification method, using a standard curve of known copy number targets (Figure S8). Standard curves were run in the same plate where possible. Otherwise, the most recent standard curve was used, and the threshold of the sample target amplification was adjusted to that of the standard curve. Target copy numbers were normalized against 18S copy numbers as a housekeeping control.

### Single-Molecule Fluorescent In Situ Hybridization (smFISH)

smFISH was performed using the RNAscope Multiplex Fluorescent Reagent Kit v2 kit (Bio-Techne) per the manufacturer’s protocol unless otherwise stated. Hybridization probes for MAP1A (Rn-MAP1A), CAMK2A (Rn-CAMK2A-C2), and positive/negative controls were ordered from the manufacturer. For MAP1A smFISH, cells were stained with Wheat Germ Agglutin (WGA) conjugated to Alexa Fluor 647 (Invitrogen) to stain the neuronal structure before mounting. For the visualization of dendritic spines, the RNAscope protocol was adapted by the addition of a gentle permeabilization step using 0.1% Triton-X for 5 min following hydrogen peroxide treatment. A 1:30 dilution of protease III was also used for 10 min. Cells were stained with Alexa Fluor 568 Phalloidin (Invitrogen) diluted 1:500 in blocking buffer (Bio-Techne) for 1 h before mounting. After all targets were amplified and conjugated with a fluorophore, the samples were counterstained with DAPI (Bio-Techne) and mounted onto microscope slides (VWR) using ProLong Gold Antifade Mountant with DAPI (Invitrogen). Three-dimensional (3D) imaging was performed on a Leica Stellaris 8 confocal microscope or a Zeiss Axio Observer widefield microscope. Deconvolution was performed after imaging using Huygens Essential software.

Images were analyzed using Fiji. To enable greater clarity in identifying dendritic spines, the contrast of the phalloidin channel was enhanced, and spines were identified as small bulbous protrusions from the dendrites. Particle analysis was used to map individual mRNA molecules to measure the localization of CAMK2A mRNA to the nearest spine. The distance to the nearest spine was measured between the centroid of the mRNA particle and the base of the nearest dendritic spine. mRNA distances from 1–2 dendritic segments (20.07 μm^2^) per cell were measured from 5 cells per condition.

### Immunocytochemistry

Immunocytochemistry was used to assess dendritic lengths and morphological changes in developing neurons. Cells were fixed using 4% PFA (Thermo Scientific) for 10 min and washed with PBS. Cells were then permeabilized with 0.1% Triton-X-100 (Thermo Scientific) in PBS for 15 min. Cells were washed in PBS and then blocked for 1 h at room temperature in 5% Bovine Serum Albumin (BSA) (Thermo Scientific) with 5% goat serum (Thermo Scientific) in PBS. MAP2 antiguinea pig primary antibody (118 004, Synaptic Systems) was diluted 1:500 in blocking buffer and added to the samples overnight at 4 °C. The next day, samples were washed in PBS and Alexa Fluor 647 goat antiguinea pig (#A-21450, Invitrogen). A secondary antibody diluted 1:1000 in blocking buffer was added to the samples for 2–3 h. Cells were washed in PBS and then mounted using ProLong Gold Antifade Mountant with DAPI (Invitrogen). Imaging was performed on a Zeiss Axio Observer widefield microscope. Images were analyzed using Fiji. The SNT toolbox was used to trace and measure dendritic lengths.^56^

### Data Analysis

Data processing and graph plotting were performed using MATLAB 2020 or Origin 2020. Normality and statistical tests were performed using GraphPad Prism 8. Outliers were identified using the Grubbs’ test (α = 0.05) and excluded from analysis. The Shapiro-Wilk test was used to test for normality before all statistical tests. A nonparametric test was used if the data failed to pass the normality test. *p* < 0.05 was considered statistically significant.

## Supplementary Material



## Notes

Polarity of neurons; Label-free and labeled sampling from live neurons; cell viability following multiple nanobiopsies from the same cell; qPCR melt curve analysis of nanobiopsies from the compartments of neurons; dendritic lengths and extent of dendritic branching of neurons between DIV 3–11; smFISH controls; Dendritic spine biopsies from the same cell to study changes in mRNA localization with stimulation; standard curves for qPCR absolute quantification; list of primer and probe sequences used in qPCR (PDF)

## Abbreviations

DEP: dielectrophoresis
LTP: long-term potentiation
smFISH: single molecule fluorescent in situ hybridization
