# Supplementary material for: Spatial and Temporal Single-Cell Profiling of RNA Compartmentalization in Neurons with Nanotweezers
Source: ACS Nano. 2025 May 6;19(19):18522–33. doi: 10.1021/acsnano.5c02056 (PMC12096465; doi:10.1021/acsnano.5c02056)
Supplement: Supplementary file 1 [file nn5c02056_si_001.pdf]

Supporting Information

Spatial and temporal single-cell profiling of RNA compartmentalization in neurons with nanotweezers

Annie Sahota[a], Binoy Paulose Nadappuram[a,b], Zoe Kwan[a], Flavie Lesept[c], Jack H. Howden[c], Suzanne Claxton[d], Josef T. Kittler[c], Michael J. Devine[e,f,], Joshua B. Edel[a,*], Aleksandar P. Ivanov[a,*].*

[a] Department of Chemistry, Imperial College London, Molecular Science Research Hub, London, W12 0BZ, United Kingdom.

[b] Department of Pure and Applied Chemistry, University of Strathclyde, Glasgow, G1 1BX, United Kingdom.

[c] Department of Neuroscience, Physiology and Pharmacology, University College London, Gower Street, London, WC1E 6BT, United Kingdom.

[d] Kinases and Brain Development Lab, The Francis Crick Institute, 1 Midland Road, London, NW1 1AT, United Kingdom.

[e] Mitochondrial Neurobiology Lab, The Francis Crick Institute, 1 Midland Road, London, NW1 1AT, United Kingdom.

[f] Department of Clinical and Movement Neurosciences, UCL Queen Square Institute of Neurology, University College London, London, WC1N 3BG, United Kingdom.

*e-mail: alex.ivanov@imperial.ac.uk; joshua.edel@imperial.ac.uk; michael.devine@crick.ac.uk

KEYWORDS: single-cell, nanotweezer, nanobiopsy, neuron, RNA, synaptic plasticity.

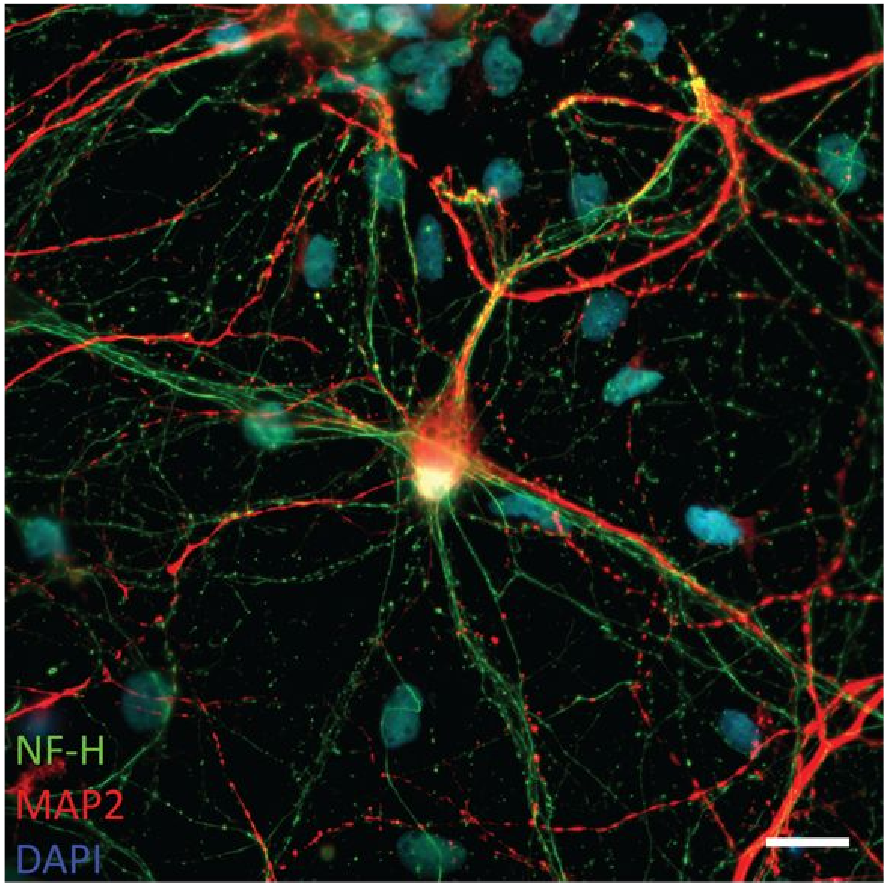

Figure S1. Polarity of neurons. Immunofluorescence staining of hippocampal neurons stained for NF-H (green) to label the axons, MAP2 (red) to label the dendrites, and DAPI (blue) to counterstain the nuclei. Scale bar = 20 μm.

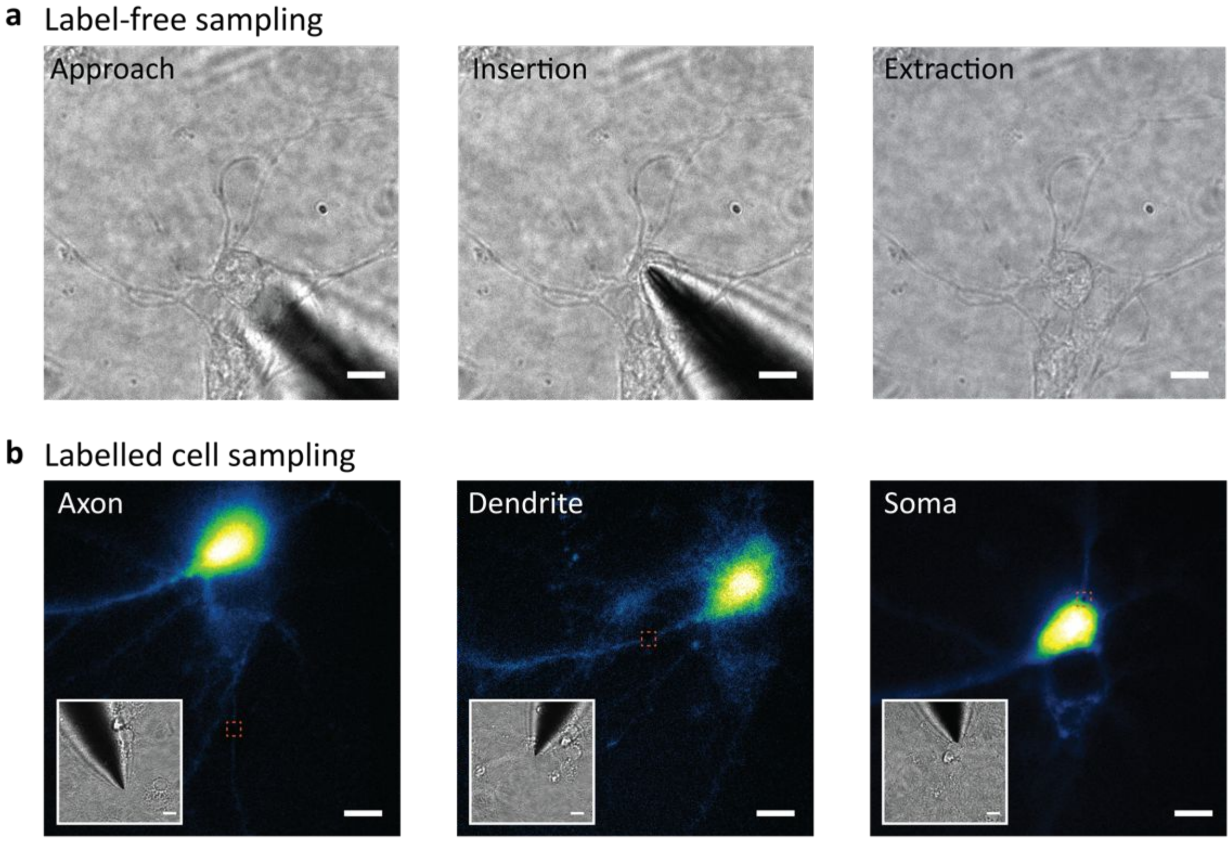

Figure S2. Label-free and labeled sampling from live neurons. a) Bright field images of the nanotweezer sampling process on label-free hippocampal neurons. b) Fluorescent images of individual biopsies from the axon, dendrite, and soma of an eGFP-expressing hippocampal neuron, corresponding to the locations mapped in Figure 2a. Insets: Corresponding bright field images of the nanotweezer during each biopsy. Scale bars = 10 µm.

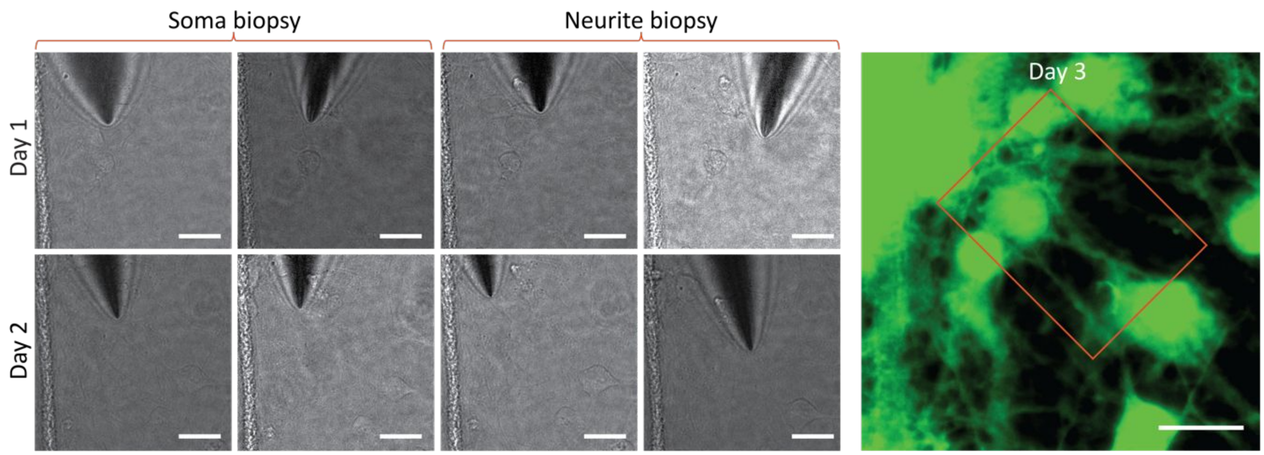

Figure S3. Cell viability following multiple nanobiopsies from the same cell. Bright-field images (left) of eight nanobiopsies taken from the same cell on two consecutive days. Calcein AM staining (right) 2 days after the first biopsy. Fluorescence indicated that the cells had intracellular esterase activity, which was used to confirm cell viability. Scale bars = 20 μm.

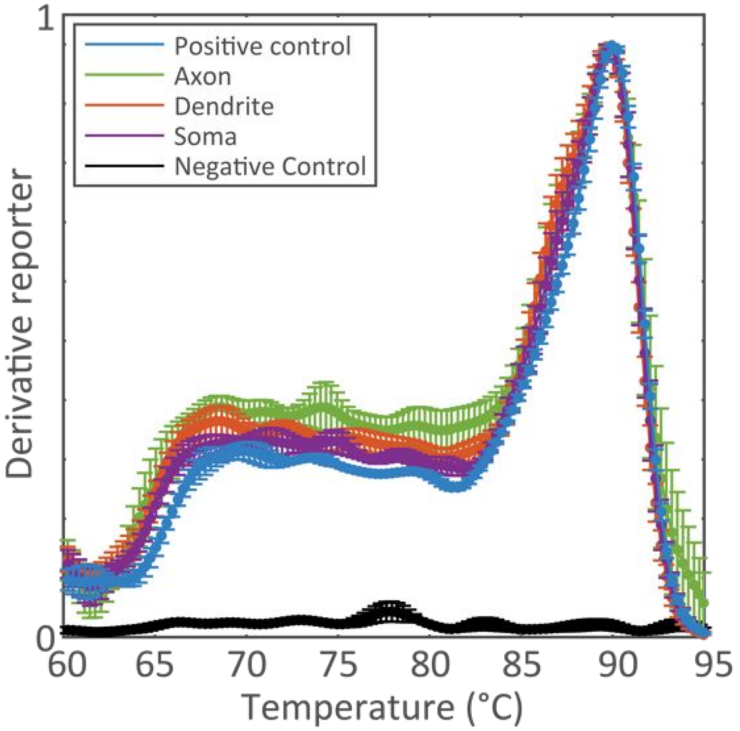

Figure S4. qPCR melt curve analysis of nanobiopsies from the compartments of neurons. Melt curve analysis corresponding to the qPCR amplification plot presented in Figure 2b for nanobiopsies from the axon, dendrites, and somata of neurons. Data presented as mean ± s.e.m (n = 3).

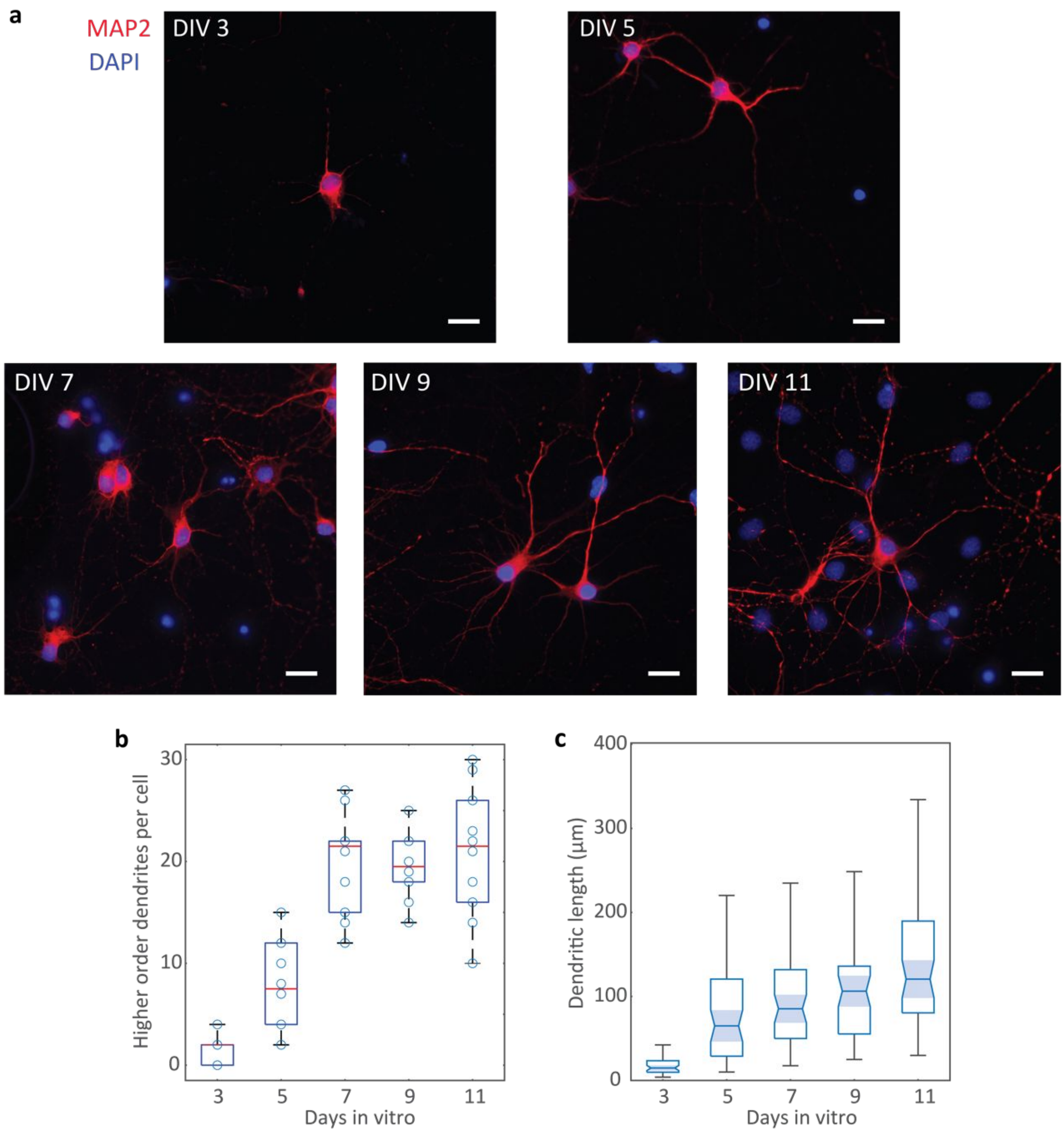

Figure S5. Dendritic lengths and extent of dendritic branching of neurons between DIV 3-11. a) Immunofluorescent images of hippocampal neurons at DIV 3, 5, 7, 9, and 11 stained for MAP2 to label the dendrites and DAPI to counterstain the nuclei. Scale bars = 20 µm. b) Boxplot of the number of higher-order dendrites (distal dendrites) per cell (n = 10 cells). c) Notched boxplot of the dendritic lengths measured in 10 cells (n = >47 dendrites). Statistical significance was

determined by a one-way ANOVA ($*P < 0.05$, $**P < 0.01$, $***P < 0.001$, $****P < 0.0001$, $^{n.s}P$ = not significant), where $P_{A,B}$ represents the significance level between groups A and B: $****P_{3,5}$, $****P_{3,7}$, $****P_{3,9}$, $****P_{3,11}$, $^{n.s}P_{5,7}$, $^{n.s}P_{5,9}$, $***P_{5,11}$, $^{n.s}P_{7,9}$, $^{n.s}P_{7,11}$, $^{n.s}P_{9,11}$. Summary statistics for boxplots: center = median; bounds of box = IQR 25th and 75th percentile; whiskers = minimum and maximum within 1.5 IQR; shaded region = notches for the variability of the medians between groups where the extremes of the notches represent q2 – 1.57(q3 – q1)/sqrt(n) and q2 + 1.57(q3 – q1)/sqrt(n), where q2 is the median (50th percentile), q1 and q3 are the 25th and 75th percentiles, respectively, and n is the number of observations. Notches that do not overlap have significantly different medians at the 5 % level.

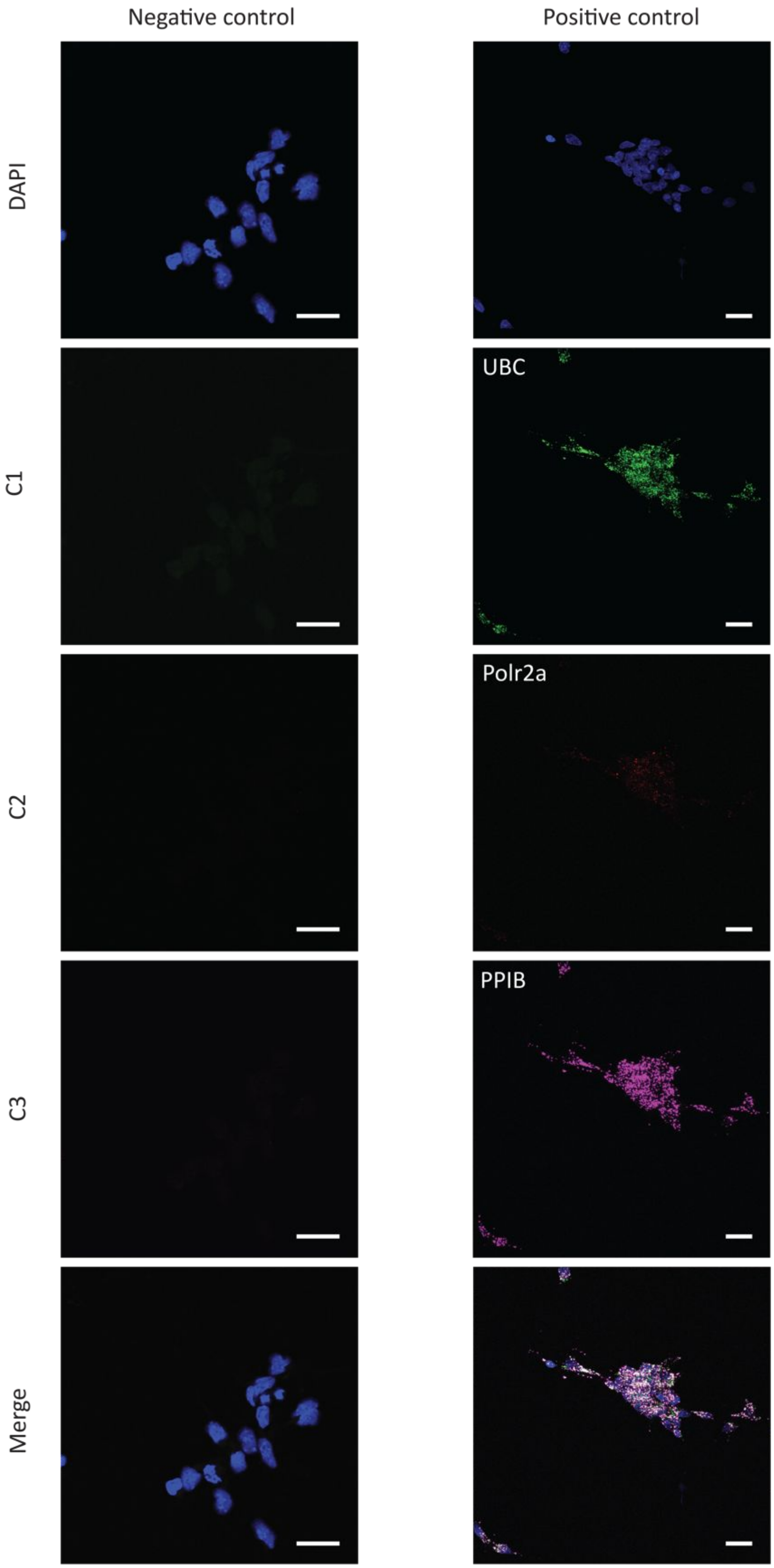
Negative control
Positive control
DAPI
C1
C2
C3
Merge
UBC
Polr2a
PPIB

Figure S6. smFISH controls. Representative images of negative (left) and positive (right) controls used to optimize protocols for smFISH using RNAscope®. The full protocol was performed for negative control probes, which do not hybridize to any RNAs, followed by nuclear counterstaining with DAPI (blue). Minimal background signal was observed. For positive controls, probes that hybridize to UBC (C1, green), Polr2a (C2, red), and PPIB (C3, magenta) RNAs were employed with the full protocol, followed by nuclear counterstaining with DAPI (blue). Distinct punctuate signals were observed in all channels that corresponded to the expected abundancies of these RNAs. Scale bars = 20 μm.

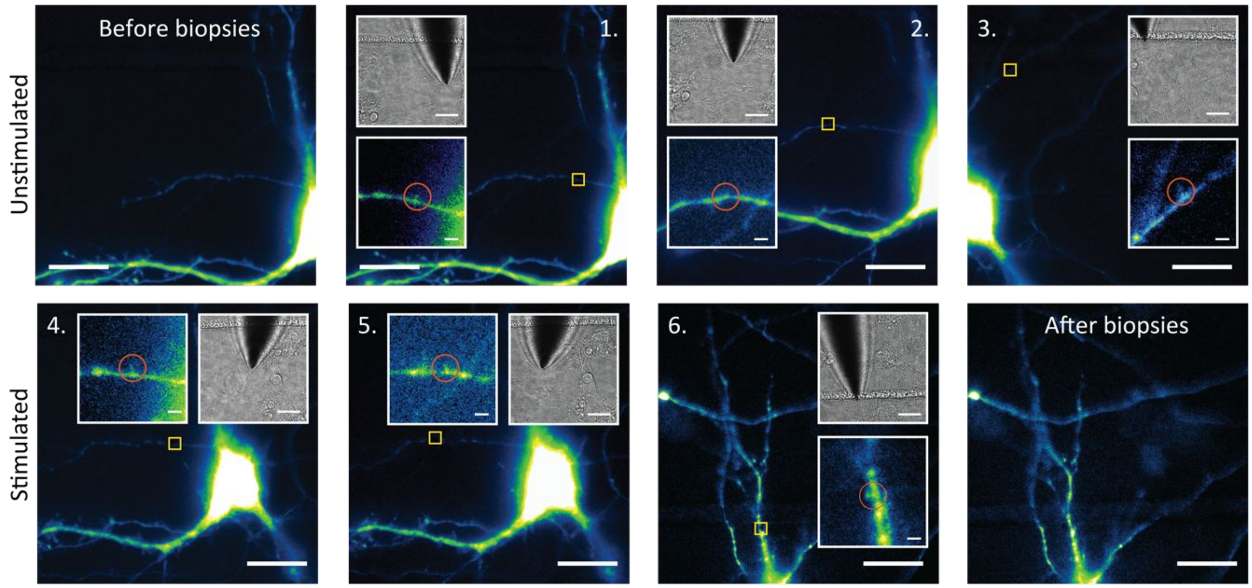

Figure S7. Dendritic spine biopsies from the same cell to study changes in mRNA localisation with stimulation. Representative images of one time-dependent stimulation study on the same neuron. Neurons were filled with eGFP to visualize the dendritic spines. Three nanobiopsies were performed at the base of the dendritic spines of the same cell before stimulation. Cells were then chemically stimulated and a further three nanobiopsies were performed on the cell at the base of the dendritic spines. Cells retained eGFP expression throughout and after biopsies, and dendritic spines remained intact. The yellow square box indicates the nanobiopsy position. Bright field inset: Corresponding bright field images of the nanotweezer inserted into the cell during nanobiopsy. Fluorescent inset: images of targeted dendritic spine regions for each nanobiopsy (orange circle). The contrast of inset images was adjusted for better spine visualization. Main scale bars = 20 μm, bright field inset scale bars = 20 μm, fluorescent inset scale bars = 2 μm.

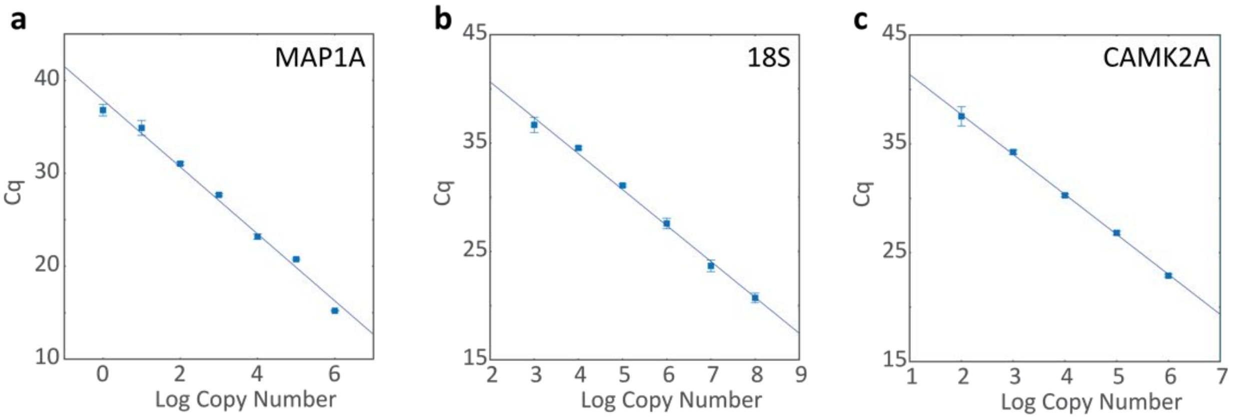

Figure S8. Standard curves for qPCR absolute quantification. Typical standard curves used for absolute quantification of the a) MAP1A, b) 18S, and c) CAMK2A content of nanobiopsy samples. Curves were constructed from serial dilutions of known copy numbers of cDNA containing the amplified region of interest. MAP1A: slope = -3.600, R^2 = 0.987, efficiency = 90%. 18S: slope = -3.314, R^2 = 0.990, efficiency = 100%. CAMK2A: slope = -3.690, R^2 = 0.995, efficiency = 87%.

Table S1. 18S copy numbers derived from intracellular nanobiopsies in Figure 2.

Cell	Soma copy number	Dendrite copy number
1	62,235	63,953
2	43,927	46,002
3		41,444
4		37,015
5	58,900	55,456
6	75,858	
7	12,458	11,330
8	12,714	
9	9,220	
10	17,256	13,297
11		12,380

Table S2. Normalized MAP1A copy numbers for dendrite-to-soma ratios in Figure 3.

DIV	Cell	Soma normalized copy numbers × 10^{-4}	Dendrite normalized copy numbers × 10^{-4}
3	1	8.407<br>6.817	2.815<br>5.297
3	2	8.922	8.066<br>4.898
3	3	7.886	6.067<br>4.169
3	4	3.211<br>3.929	2.699<br>2.842
3	5	5.049<br>7.689<br>6.795	2.848
3	6	5.824	1.067
5	7	6.486	3.4<br>8.247
5	8	2.3715<br>0.2153<br>9.7373<br>36.3881	0.9231<br>2.14<br>6.8778
5	9	3.004<br>1.679	1.861
5	10	1.732<br>1.618	1.706<br>5.778
5	11	1.678	0.4958
7	12	0.6886<br>0.7717	0.4487<br>1.141
7	13	1.073<br>2.935<br>1.093	1.145
7	14	31.47<br>8.421	18.83<br>2.257

7	15	3.759<br>19.4	4.664<br>9.866<br>1.275
7	16	4.652	1.257<br>2.509
7	17	8.087	5.435
9	18	0<br>19.74	15.26<br>0
9	19	0<br>24.27	0<br>6.764
9	20	7.468	4.517
9	21	2.863	1.713
9	22	16.67<br>24.15	16.56
9	23	7.224<br>6.358<br>0	8.56<br>7.142<br>7.58<br>0
11	24	1.311<br>2.332<br>2.858<br>3.915	1.832<br>4.988<br>2.045<br>3.899
11	25	8.044<br>10.28	4.794<br>7.81
11	26	22.09	16.23
11	27	4.423<br>3.64	4.711
11	28	16.49	14.84

Table S3. Normalized CAMK2A copy numbers for single-cell tracking in Figure 4.

Cell	Treatment	Normalized CAMK2A copy numbers before treatment × 10^{-3}	Normalized CAMK2A copy numbers after treatment × 10^{-3}
1	LTP	6.594<br>10.07	15.96<br>22.74
2	LTP	4.449<br>7.267<br>7.13	46.17<br>6.566<br>27.63
3	LTP	0<br>6.055<br>2.16	17.81<br>0<br>24.31
4	LTP	0.07761<br>0.2466<br>0.06876	0<br>0.5743<br>0.4727
5	LTP	1.631<br>1.451<br>0	0<br>7.429<br>0.248
6	Control	19.93<br>8.747<br>31.24	8.063<br>13.18<br>9.268
7	Control	3.977<br>2.39	8.197<br>4.702
8	Control	0<br>46.8<br>0	57.55<br>0<br>6.372
9	Control	0.4186<br>26.29<br>0	10.6<br>2.131<br>4.601

Table S4. List of primer and probe sequences used in qPCR.

Target	Forward primer	Reverse primer	Probe
18S (dye-based)	GGTAACCCGTTGAACCCCAT	TTCGACCGTCTTCTCAGCG	N/A
18S	CCAGTAAGTGCGGGTCATAA	GGCCTCACTAAACCATCCAA	TGCGTTGATTAAGTCCCTGCCCTT
MAP1A (Assay Rn.PT.58.9611736.gs, Integrated DNA Technologies)	TGGAAATGACCCTGCCAATG	TCTGCTGATACCACTCACGA	TCAGGTGACTCTGATTCCCACTCATGA
CAMK2A (Assay ID Rn.PT.58.18655830, Integrated DNA Technologies)	GTGCTACACTGTCACCAGATG	TGCTCTCCCTCAACCTCTATG	CGCGACCTGAAGCCTGAGAATCTG

All primers/probes were used in probe-based qPCR unless stated as dye-based. Assay numbers are listed for primers/probes purchased commercially.
